# ‘Both parents should care for babies’: A cross-sectional, cross-cultural comparison of adolescents’ breastfeeding intentions, and the influence of shared-parenting beliefs

**DOI:** 10.1186/s12884-017-1372-y

**Published:** 2017-06-29

**Authors:** Vivien Swanson, Leena Hannula, Linda Eriksson, Malin Häggkvist Wallin, Joan Strutton

**Affiliations:** 1 School of Natural Sciences, University of Stirling, Stirling, FK9 4LA Scotland; 2 Faculty of Health Care and Nursing, Helsinki Metropolia University of Applied Sciences, Helsinki, Finland; 3 School of Health in Social Science, University of Edinburgh Medical School, Teviot Place, Edinburgh, EH8 9AG Scotland; 4 Psykologisk Institutt, UiO/University of Oslo, Boks 1072 Blindern, 0316 Oslo, Norway; 5 Psychology and Counselling, Texas A & M University – Central Texas, Killeen, TX 756-49 USA

**Keywords:** Adolescents, Parenting, Breastfeeding, Cross-cultural comparison, Theory of planned behaviour

## Abstract

**Background:**

Many young men and women expect to co-parent their newborn infant. This may have a positive or negative impact on decisions to breastfeed, which is an important health behaviour, influenced by cultural and psycho-social norms. We investigated the relationship between shared parenting, infant feeding beliefs and intentions in male and female (non-parent) adolescents, comparing Nordic countries (Sweden, Norway, Finland) with high breastfeeding rates with others with low rates (Scotland, USA).

**Methods:**

We utilised cross-sectional surveys of male and female adolescents (*n* = 1064, age 12–18) administered directly in schools or via the internet. We assessed attitudes to breast and formula feeding and shared parenting, using a Theory of Planned Behaviour framework, assessing beliefs, attitudes, norms and control as predictors of intention.

**Results:**

Male and female adolescents’ breastfeeding intentions varied in line with national cultural norms. Young people from Nordic countries (high breastfeeding rates) were significantly more likely to intend to breastfeed than those from Scotland or the USA (low breastfeeding rates). Positive beliefs about breastfeeding, norms and ‘exposure’ to breastfeeding and feeding confidence were consistently stronger in Nordic countries, whereas young people in Scotland had more positive beliefs, norms and ‘exposure’ to formula feeding. Differences in parenting beliefs, norms and confidence were less consistent. In logistic regression, cultural group, positive breastfeeding beliefs and exposure, norms, and shared parenting beliefs were significant predictors of breastfeeding feeding intention.

**Conclusions:**

Positive beliefs about shared parenting and equal gender norms were related to future breastfeeding intentions for female and male adolescents. Health education programmes for young people could encourage positive breastfeeding choices by considering how this would fit with young people’s ideal parenting roles, and by emphasising benefits of complementary maternal and paternal roles in breastfeeding newborn infants.

## Background

Fathers’ involvement in early parenting in western cultures has increased steadily [1, 2] and traditional gender roles, including taking part in a caring role for newborn infants, have merged [3]. Family structures are more fluid, many women work outside the home and aim to work post-childbirth. Shared parenting (co-parenting) is becoming normal, and the notion of the involved, caring father becoming ‘culturally embedded’ [4]. Family systems research suggests shared parenting is beneficial for the mother/partner/infant triad, leading to better parental care and satisfaction and more positive psychological outcomes for the child [5].

An important early decision for many new parents is around infant feeding – whether to breastfeed or formula feed. Breastfeeding has clear health advantages for the infant [6, 7] including better long term cognitive outcomes [8] and reduced childhood obesity [9]. Positive maternal health outcomes include reduced risk of type 2 diabetes, some cancers [7] and post-partum depression [10]. Promoting initiation and maintenance of breastfeeding for the first six months of life [11] is an important health improvement target. Internationally breastfeeding initiation rates have improved, but not duration, despite health promotion efforts [12]. A Scottish study found little change in a 10 year follow-up of young people’s attitudes and intentions [unpublished observation, Swanson, 2015].

### Cultural differences

There are notable national differences in breastfeeding initiation and maintenance. In Scotland, 74% of new mothers initiate breastfeeding [12], comparing unfavourably with England (83%). Northern European countries, including Norway, Finland and Sweden, have initiation rates around 98% [13–15]. Rates for the USA were 77% in 2013, with significant differences between states [16]. Understanding and measuring cultural difference and its impact on health behaviours is complex, with as many cultural variations within countries as between them [17]. Cultural influences operate at different levels, including individual psychosocial factors, inter-generational transmission within families, social and community influences [18], socio-economic status (SES) and ethnic differences [17]. National policies supporting parents, including maternity and paternity leave and hospital and community healthcare are important [19, 20]. This study aims to learn from studying the impact of cultural norms on individual psychosocial influences on adolescents’ infant feeding and parenting beliefs, examining shared sets of beliefs, meanings and values (social norms) which are socially transmitted guide behaviour, and shaped by lived experiences [21], comparing countries with high (Sweden, Finland, Norway) and low (Scotland, USA) breastfeeding initiation rates.

### Infant feeding and parenting

Infant feeding decisions are often made early, before pregnancy [22]. Discussing these choices with young men and women (pre-parenting) is important, and school-based interventions promoting breastfeeding are valuable [19, 23, 24] (but with little evidence of long-term influences on behaviour). Breastfeeding is often discussed in the context of sex education, child development, or nutrition [25] which young people (and teachers) may find embarrassing, or seemingly irrelevant. For more lasting cultural change, a different approach is needed. Breastfeeding choices may appear more relevant to young men and women when discussed in the broader context of their desired parenting roles and intentions [23]. However, it is unclear how shared parenting beliefs relate to adolescents’ breastfeeding intentions. Since only the mother can breastfeed, partners may feel excluded from early parenting and ‘bonding’ with the infant [26], affecting both parents’ decision making [27]. Partners’ support for the breastfeeding mother is important for maintaining breastfeeding for recommended periods [28, 29]. A decision to formula-feed may reflect preference for more childcare involvement from the father [30] or lack of breastfeeding support.

### Theoretical background

The theory of planned behaviour (TPB) [31] is useful for assessing the influence of socio-cognitive factors [32], and has been applied to infant feeding in new parents, [33, 34], parents-to-be [35], and non-pregnant adolescents [24, 36]. Demographic factors, including ethnicity, age, sex and SES are important confounders [37] when included as first-line predictors [38].

### Aims

We compared (non-parent) male and female adolescents’ shared parenting beliefs, norms, confidence and intentions and breastfeeding and formula feeding intentions, in countries with contrasting breastfeeding rates. Research questions were:

1. How do (non-parent) male and female adolescents’ infant feeding and parenting intentions for newborn infants vary in countries with high and low breastfeeding rates?
2. How do TPB psychosocial factors related to a) breastfeeding or formula feeding (beliefs, norms and confidence) and b) parenting (attitudes, gender roles norms and confidence) vary for adolescents in countries with high and low breastfeeding rates?
3. To what extent do a) breastfeeding and formula feeding beliefs and b) parenting beliefs, influence adolescents’ future infant feeding intentions?

## Methods

Cross-sectional surveys were conducted in a 12 month period in 2013–14 in Scotland, the USA and three Nordic countries. Samples represented specific socio-geographical areas rather than countries as a whole.

### Recruitment of participants

In central Scotland, three schools in urban areas with a broad socio-economic mix were invited to participate with prior permission from the Local Education Authority. In Sweden, two schools in the Mälardalen region (mixed SES metropolitan area around Stockholm) were contacted and agreed to participate. Parents’ permission was sought on an ‘opt-out’ basis for pupils. A hard copy letter invited parents to respond by returning a slip to school to exclude their child; no opt-out requests were received in either country. With consent, paper-based surveys were completed and placed in sealed envelopes by participants, collected and returned to researchers unopened by teachers (Scotland, Sweden).

Identical on-line surveys [39], were distributed by teachers in schools in Norway and the USA. In Norway, 3 schools around Oslo, (mixed SES) were recruited by personal contact. Parents first received hard-copy opt-out letters as above. Pupils later completed on-line surveys in school settings, having consented to take part. In the USA, permission was requested from school district administrators in Texas. A survey link was then forwarded to parents to first seek consent and then pass on to the participant.

In Finland, a ‘snowballing’ method and a popular internet site for young people (http://irc-galleria.net) recruited school aged participants in the urban Helsinki area.

For Sweden, Norway and Finland, the questionnaire was translated and back-translated into the first language using a translation service, and checked for face validity. In the USA, some terminology was changed (e.g. ‘nappies’ (UK) to ‘diapers’ (USA)).

Most (96%) of US participant responses came from 14 urban counties in Texas, (77% breastfeeding initiation, approximating the US national average [16]). Others (4%) came from Ohio, Michigan, and Arkansas.

Participants were school pupils aged 12–18. Sample size was estimated using a priori power analysis with G*Power [40]. Using linear regression with 13 predictors, medium ES, α = .01, 90% power, we required 192 participants. We recruited additional participants to allow for missing data, and to enable sub-group analysis.

### Ethical approval

The study was given ethical approval by the host institution’s University Psychology Ethics of Research Committee, including approval to collect data in Sweden, Finland and Norway subject to local permissions and with local school approval from Scottish education authorities. Authorities deemed this sufficient to negate the need for further institutional ethical review in Sweden and Norway, where additional school-based permissions were then obtained, and in Finland, where additional permission was given by a national social networking site adminstrator. The Internal Review Board of Texas A&M University (Central) gave USA approval.

All participants obtained parental consent to participate, using slightly different methods (see above). Immediately before completion the voluntary nature of the questionnaire was emphasised to potential participants who were given the opportunity not to participate. The on-line version included mandatory check boxes indicating prior parental consent and participant consent, before the questionnaire would open. It was incentivised for individual participants (young people) offering an on-line voucher for every 25th response. Inclusion in this draw, (via their email) was voluntary.

### Demographics

Age was coded into 3 categories: 12–13, 14–15, 16–18. Mothers’ highest level of education provided a proxy for SES [12, 41]. In Scotland we recorded four levels; ‘standard grades’ (national qualification, age 15–16), ‘highers’ (national qualification, age 16–18), vocational qualifications and university degree. Equivalents were determined for USA and Nordic countries.

### Study variables

The main study outcomes was infant feeding intentions. Predictors were infant feeding beliefs, measured in a TPB framework, measuring beliefs/attitudes, norms, perceived behavioural control and parenting beliefs, including attitudes, gender norms and confidence. Scales’ reliability was established using Cronbach’s alpha throughout (α).

### Infant feeding

For consistency, we defined infant feeding at the start of questionnaires: Breast-feeding – baby gets milk from the mother’s breast; Formula-feeding – baby gets formula/powdered milk from a bottle. Combined feeding – baby gets both breast milk from the mother and formula milk from a bottle.

### Own feeding experience

Participants were asked how they had been fed themselves as a newborn; (options: ‘breast-fed’, ‘formula-fed’, ‘combined-fed’, ‘don’t know’). We combined the ‘breast-fed’ and ‘combined-fed’ responses, and excluded ‘don’t know’ to create a dichotomous variable representing ‘any breast-feeding’ vs. ‘no breast-feeding’.

### Future feeding intentions

We asked how they wished to feed their baby if they became a parent, with fixed choice options as above, creating a dichotomous ‘any breast-feeding’ variable for analysis.

### Infant feeding behavioural beliefs

Items previously used with new parents and adolescents in Scotland [34, 36] measured behavioural beliefs about breastfeeding and formula feeding, avoiding social desirability bias towards breastfeeding as a desired health behaviour. Initially, thirteen items were scored on a Likert scale: 1 ‘strongly disagree’ to 7 ‘strongly agree’. PCA with varimax rotation identified a forced 2 factor solution with four variables eliminated (using cut-off 0.4, [42]) since they did not load onto either factor. Factors represented positive (4 items, 19.6% of variance) and negative beliefs about breastfeeding (5 items, 27.8% of variance). Positive beliefs included: ‘breastfeeding leads to a close bond between mother and baby’, ‘…helps get the mother’s figure back to normal’, ‘…is a natural way of feeding babies’, and ‘formula feeding is expensive’ (α = .59). Negative beliefs included: ‘formula feeding makes it easier for mother to go back to work’, ‘breastfeeding is embarrassing for the mother’, ‘breastfeeding spoils the shape of the mother’s breasts’, ‘breastfeeding limits the mother’s social life’, and ‘breastfeeding can be uncomfortable for the mother’ (α = .67). Reliability of factor solutions remained consistent in each cultural group.

### Social norms

We assessed descriptive (what other people do) and injunctive (subjective) norms (perceived social pressure) [43].

### Descriptive norm

‘Exposure’ to breastfeeding and formula feeding was calculated, from 4 social referent groups at variable social distance [36] (family member, friend, someone you don’t know, someone on television), e.g.: ‘how often have you seen a close family member breastfeeding a baby?’, scored 1–4; ‘never’, ‘1–2 times’, ‘3–10 times’, ‘more than 10 times’, summed to represent total exposure to breastfeeding and formula feeding (range 4–16).

### Injunctive norms

Subjective norms (SN) were measured with 2 single items using a 7 point Likert scale with anchors as above: ‘People important to me would want me to breastfeed/formula feed my baby’.

### Infant feeding confidence

Two summed items measured perceived control for each parent: ‘For the mother, breastfeeding a new baby would be..’; ‘For the father, helping to feed a new baby would be..’ scored on a 7 point Likert scale from ‘very difficult’ to ‘very easy’, (α = .52).

### Parenting

#### Parenting intentions

We asked if participants intended becoming a parent in the future (yes =3, not sure = 2, no = 1), combining the last two groups, creating a binary variable.

#### Parenting attitudes

Two summed items measured attitudes to being a parent of a newborn; ‘overall being the parent of a new baby would be..’ rated on a 7 point scale from ‘very unpleasant’ to ‘very pleasant’, and ‘very bad’ to ‘very good’, with mid-points at ‘neither’. High scores represented more positive attitudes, (α = .85).

#### Parenting confidence

Two items measured confidence; ‘overall being a parent of a new baby would be…’, rated on a seven point scale from ‘very difficult’ to ‘very easy’, and ‘how confident would you be about being the parent of a new baby?’, from ‘very unconfident’ to ‘very confident’. Higher scores represented more confidence (α = .55).

#### Attitudes to shared parenting

Eight items measured attitudes, adapting and augmenting a 3 item ‘Equality in feeding’ factor from a Finnish measure of parents’ breastfeeding attitudes [35], making it applicable to non-parents. Items included; ‘baby receives breast milk’, ‘keep up with your friends’, ‘decide about feeding method’, ‘both can feed the baby’, ‘spend the same amount of time with the baby’, ‘share baby’s care equally’, ‘have time to yourself’, and ‘have time alone with the baby’. Items were scored on a 5 point Likert scale, anchors from ‘not at all important’ to ‘extremely important’. High scores reflected more positive shared parenting attitudes. Principal Components Analysis (PCA) with varimax rotation led us to discard 2 items: ‘baby receives breastmilk’ and ‘have time alone with the baby’. We identified 2 factors with eigenvalues over 1: ‘Equal parenting’ (4 items, α = .72, 38% of variance) and ‘Parental independence’ (2 items, α = .67, 23% of variance), providing better reliability than the total (α = .63). Reliability of factor solution and variance predicted was similar in each cultural group.

#### Gender role norms

We assessed gender role norms for parents’ participation in common tasks for newborns asking; ‘if both parents are around, how much do you think the mother or father *should do* these things for their newborn baby’? Eight tasks included: ‘getting up in the night to feed the baby’, ‘changing diapers’, ‘feeding the baby’, ‘playing with baby’, ‘taking baby out’, ‘taking baby to nursery’, ‘soothing baby when it cries’, and ‘babysitting’, scored from ‘mother does all of the time’ (score − 2) to ‘father does all of the time’ (score + 2), mid-point ‘both parents do equally’ (score 0). Higher scores reflected more paternal involvement. A total ‘gender role’ score summed these variables (α = .75).

#### Analysis

Data from Nordic participants were combined, having checked for significant differences between countries. There were no differences in feeding intentions and the overall pattern was homogeneous. Variables were checked for normality and patterns of missing data with no adjustments required. Totals reported in tables and text vary slightly due to some missing data, however missing data percentages were calculated for study variables and were generally low (between 0.5 and 6.0%). Proportions in categorical variables were examined using χ^2^ tests, and relationships using Pearson correlations. National group and sex differences were estimated using t-tests, one-way or factorial ANOVA. Age and sex were covariates. Planned hierarchical multiple regression predicted intended infant feeding outcomes from national group, demographic confounders, TPB infant feeding and parenting variables. Standardised beta values (95% CI for B) assessed predictors. Effect size (ES) was estimated using r, Cohen’s D, or partial η^2^ as appropriate.

## Results

Table 1 shows participants’ demographic characteristics. The Nordic sample included 83, 47% males, 95, 53% females from Sweden; 40, 36% males, 71, 64% females from Norway; and 10, 10% males, 88, 89% females from Finland. Scotland had a higher proportion of males, and a younger age profile. Mother’s education level was lower, reflecting lower SES in the Scottish sample.

**Table 1:** Demographic Characteristics of participants from Scotland, Nordic Countries and the USA

	Scotland (*n* = 507, 48%)	Nordic (*n* = 387, 36%)	USA (*n* = 170, 16%)	Comparisons	Sig.
Female	254 (50.4)	253(65.7%)	105 (61.8%)	χ^2^ (df2) = 22.3	*p* < 0.001
Male	250 (49.6)	132 (34.3%)	65 (38.2%)		
Mean Age	14.1 (1.4)	15.4 (1.3)	15.7 (1.5)	F(2,1060) = 95.19,	*p* < 0.001
Mother’s education level^a^	2.25 (1.1)	2.95 (1.1)	3.0 (.83)	F (2, 843) = 46.7	*p* < 0.001

^a^Measured on a 4 point scale where 4 is high

We were interested in how male and female adolescents’ infant feeding and parenting intentions for newborn infants varied between countries with high and low breastfeeding rates. Table 2 shows how participants were fed themselves and their own infant feeding intentions by national group.

**Table 2:** Participants’ Own Feeding Method and Intention, by National Group

	Breast-fed	Formula-Fed	Combined	Don’t know	Total (100%)
	Own feeding method				
Scotland	112 (22%)	193 (39%)	53 (11%)	143 (28%)	501
Nordic	260 (67%)	19 (5%)	43 (11%)	63 (16%)	385
USA	41 (24%)	36 (21%)	55 (32%)	38 (22%)	170
	Future feeding intentions				
Scotland	119 (24%)	136 (27%)	106 (21%)	135 (27%)	496
Nordic	207 (54%)	6 (2%)	113 (29%)	60 (15%)	386
USA	52 (32%)	20 (12%)	69 (41%)	29 (17%)	170

There were strong differences in how they had been fed (χ^2^(6) = 290.6, *p* < .001). Many more Nordic participants had been breastfed, whereas more Scottish participants had been formula fed. Far fewer Nordic participants (16%) did not know how they had been fed than Scottish (28%) and US participants (22%). Feeding intentions also varied significantly by national group (χ^2^ (6) = 184.5, *p* < .001), mirroring national initiation rates.

Using dichotomous (breast-fed or not) outcomes, we found highly significant associations between how they had been fed and future intentions for Scottish (χ^2^(1) =52.3, *p* < .001); Nordic (χ^2^(1) = 24.6, *p* < .001); and USA (χ^2^ (1) = 19.4, *p* < .001) participants.

There were no significant age differences in how participants had been fed or feeding intention. In Scotland, slightly more males (79, 52%) than females (84, 41%) were breastfed (χ^2^ (1) = 4.09, *p* = .04), and more males (117, 67%) intended their own baby would be breastfed than females (107, 58%), (χ^2^ (1) = 3.38, *p* = .07), with no differences for Nordic or US participants.

### Parenting intention

Taking the whole sample, most participants (662, 63%) ‘definitely’ intended to be a parent, a third (346, 32.4%) were unsure, and few (51, 4.8%) definitely did not want to become a parent. More females (66%) than males (58%) definitely wanted to become a parent, whereas more males (38%) were unsure than females (29%). More females (5.3%) than males (4.0%) ‘definitely’ didn’t want to become a parent (χ^2^ (2) = 8.9, *p* = .012). There were striking differences by country. Only 41% of participants in Scotland ‘definitely’ intended to become a parent, compared with 71% in the USA and 71% in Nordic countries (χ^2^ (4) = 41.8, *p* < .001).

We investigated differences in TPB psychosocial factors (beliefs, norms and confidence) related to both breastfeeding or formula feeding and parenting, comparing adolescents in countries with high and low breastfeeding rates. Table 3 shows mean comparisons for TPB infant feeding variables by national group, age group and sex, using 3x3x2 factorial ANOVA, and correlations for the whole sample. There were highly significant national effects for all infant feeding variables. Scottish participants had lower positive and higher negative breastfeeding beliefs, less breastfeeding exposure, less positive breastfeeding norms, more positive formula feeding norms and lower breastfeeding confidence. USA participants had most positive and least negative breastfeeding beliefs and most formula feeding exposure. Effect sizes (partial η^2^) were generally small.

**Table 3:** ANOVA Comparisons and Effect Sizes for National Group, Age Group and Sex, and whole sample Pearson Correlations for Infant Feeding Variables

	Variable	ANOVA Comparisons			Pearson Correlations						
		National Group	Age Group	Sex	1	2	3	4	5	6	7
1	Positive BF beliefs	USA > Nordic > Scot F = 4.97** (η^2^ = .01)	2 > 3 > 1 F = 1.04 (η^2^ = .002)	F > M F = 1.68 (η^2^ = .002)	1.0						
2	Negative BF beliefs	Scot > Nordic > USA F = 7.09** (η^2^ = .01)	2 > 3 > 1 F = .94 (η^2^ = .002)	F > M F = .3.08 (η^2^ = .003)	.09**	1.0					
3	BF exposure	Nordic > USA > Scot F = 156.9*** (η^2^ = .23)	3 > 2 > 1 F = 8.56*** (η^2^ = .02)	F > M F = 10.17*** (η^2^ = .01)	.21**	−.04	1.0				
4	FF exposure	USA > Scot > Nordic F = 48.46*** (η^2^ = .09)	3 > 2 > 1 F = 2.59 (η^2^ = .001)	F > M F = 16.87*** (η^2^ = .02)	.07*	.13**	.13**	1.0			
5	BF Subj. norm	Nordic > USA > Scot F = 4.89** (η^2^ = .01)	2 > 3 > 1 F = 1.68 (η^2^ = .003)	F > M F = 6.63* (η^2^ = .007)	.41***	.02	.21**	−.04	1.0		
6	FF Subj. norm	Scot > USA > Nordic F = 17.39*** (η^2^ = .03)	2 > 1 > 3 F = 1.60 (η^2^ = .003)	M > F F = .30 (η^2^ = .001)	−.20	.28**	−.19**	.13**	−.14**	1.0	
7	Feeding confidence	Nordic > USA > Scot F = 14.93*** (η^2^ = .03)	3 > 2 > 1 F = .05 (η^2^ = .0001)	F > M F = .67 (η^2^ = .001)	.18***	−.16***	.12***	−.03	.11**	−.03	1.0

*Scot* Scotland, *Nordic* Finland, Sweden, Norway; Age Groups 1 = 12–13; 2 = 14–15; 3 = 16–18; Sex: *F* Female, *M* Male; Partial η^2^ shown as η^2^; *BF* Breastfeeding; *FF* Formula Feeding Subj. norm = subjective norm ***p < .001, **p < .01, *p < .05

There were no age differences apart from breastfeeding exposure, where older groups had significantly more exposure. Sex effects were significant for breastfeeding and formula feeding exposure and breastfeeding norms. Females had higher mean scores for all variables apart from formula feeding norms. Table 3 shows positive breastfeeding beliefs were positively correlated with other feeding variables. Norms and exposure were positively related and more positive beliefs and norms indicated more infant feeding confidence.

#### Parenting beliefs

There were remarkably few sex differences in desired roles. Modal score for individual gender role items was zero, (both parents do equally), although item means were all negative, indicating most (both male and female) participants thought mothers should play a greater role in each individual behaviour, particularly feeding (overall mean − 0.38, SD = 0.63) and soothing infants (mean = −0.21, SD = 0.55). Males were significantly more likely than females to suggest the mother should change diapers (t(1053) = 6.78, *p* = .001, d = .42), soothe the baby (t(1044) = 3.34, *p* = .001, d = .21) and babysit (t(1044) =2.6, *p* = .011, d = .16).

Table 4 shows mean comparisons for parenting variables by national group (Scotland, Nordic, USA), age group (12–13; 14–15; 16–18) and sex (M/F), and whole-sample correlations. A 3x3x2 factorial ANOVA showed significant national differences in parenting attitudes, shared parenting, and parental independence, but not gender roles or parenting confidence. Adolescents in Scotland had most positive attitudes, whereas those in the USA had higher shared parenting scores. Nordic participants had higher scores on parental independence, gender roles and parenting confidence. The only significant age group differences were in relation to gender roles. Age group differences were variable, younger participants had more positive attitudes, confidence and gender roles. Females recorded more positive parenting attitudes and gender roles. Parenting variables were significantly inter-correlated, with small effect sizes.

**Table 4:** ANOVA Comparisons and Effect Sizes for National Group, Age Group and Sex, and whole sample Pearson Correlations for Parenting Variables

	Variable	ANOVA Comparisons			Pearson Correlations				
		National Group	Age group	Sex	1	2	3	4	5
1	Parenting attitudes	Scot > Nordic > USA F = 13.32*** (η^2^ = .03)	1 > 3 > 2 F = 2.10 (η^2^ = .004)	F > M F = 6.94** (η^2^ = .007)	1.0				
2	Shared parenting	USA > Scot > Nordic F = 11.15*** (η^2^ = .02)	2 > 3 > 1 F = .40 (η^2^ = .001)	F > M F = .33 (η^2^ = .0001)	.13***	1.0			
3	Parental independence	Nordic > Scot > USA F = 75.41*** (η^2^ = .13)	2 > 3 > 1 F = .01 (η^2^ = .0001)	M > F F = .02 (η^2^ = .0001)	−.09**	.12***	1.0		
4	Gender roles	Nordic > Scot > USA F = 2.76 (η^2^ = .005)	1 > 2 > 3 F = 4.10* (η^2^ = .008)	F > M F = 4.33* (η^2^ = .004)	.15**	.15**	.07*	1.0	
5	Parenting confidence	Nordic > Scot > USA F = 2.64 (η^2^ = .005)	1 > 3 > 2 F = .21 (η^2^ = .001)	M > F F = .99 (η^2^ = .001)	.48***	.11**	−.05	.02	1.0

*Scot* Scotland, *Nordic* Finland, Sweden, Norway; Age Groups 1 = 12–13; 2 = 14–15; 3 = 16–18; Sex: *F* Female, *M* Male; Partial η^2^ shown as η^2^; *BF* Breastfeeding, *FF* Formula Feeding ***p < .001, **p < .01, *p < .05

In a hierarchical binomial logistic regression, we investigated whether infant feeding beliefs, norms and confidence, and similar parenting beliefs, gender norms and confidence, added variance to predicted future intention (breastfeed/combined feed (642, 78%) vs. don’t know/not breastfeed (177, 22%). The first step was national group, dummy coded with Scotland as reference. Demographic factors (age and sex) were entered next as potential confounders. TPB infant feeding variables (behavioural beliefs, norms, confidence) were entered third. Perceived parenting variables were the final step. Prediction success of the constant-only model was 78, and 84.5% for the full model, showing improvement (94.5% breastfeeding, 48% not breastfeeding). The Hosmer-Lomershow goodness-of-fit test for the final model was non-significant suggesting a good fit (χ^2^ (8) = 6.4, *p* = .60). This model (at Step 4) was significant, χ^2^(16) = 259.5, *p* < .001. Estimated R^2^ (Nagelkerke) was 0.48 indicating a medium-sized relationship. There was a large effect of national group (Wald test, t(df2) =26.8, *p* < .001). Nordic participants were 18 times more likely to intend to breastfeed than Scottish participants (OR 17.71) (although precision of this estimate was poor, evidenced by the large 95%CI). In the final model, (Table 5) positive breastfeeding beliefs and norms (breastfeeding exposure, formula feeding exposure, breastfeeding SN, formula feeding SN) were significant predictors, as were positive attitudes to shared parenting (OR 1.09) and parenting independence (OR 1.16).

**Table 5:** Logistic regression analysis predicting Infant Feeding Intention from Demographic, TPB Variables and Parenting Variables for the whole sample

		Unstandardized B (SE)	Β (Exp B)	95% CI for Odds Ratio	
				Lower	Upper
Constant		−6.0 (1.65)			
Step 1: Cultural Group	Nordic^a^	2.89 (.59)	18.07***	5.70	57.18
	USA	−.28 (.35)	.76	.37	1.50
Step 2: Demographics	Age	.15 (.08)	1.15	.98	1.35
	Sex^b^	.40 (.25)	1.49	.91	2.40
Step 3: TPB Infant feeding	Breastfeeding Beliefs (+ve)	.16 (.04)	1.17***	1.09	1.27
	Breastfeeding beliefs (−ve)	−.04 (.03)	.96	.91	1.01
	BF Exposure	.13 (.06)	1.14*	1.01	1.27
	FF Exposure	−.10 (.04)	.90*	.82	.97
	BF Subj. norm	.24 (.09)	1.26**	1.07	1.49
	FF Subj. norm	−.34 (.10)	.71**	.58	.87
	Feeding confidence	.10 (.06)	1.10	.99	1.23
Step 4: Parenting	Parenting attitude	.04 (.05)	1.04	.95	1.15
	Shared parenting	.09 (.04)	1.09*	1.00	1.19
	Independence	.14 (.06)	1.16*	1.17	1.52
	Gender norms	−.01 (.05)	1.04	.95	1.15
	Parenting Confidence	−.10 (.05)	.90	.82	1.01

B and β values presented are from the final step of the regression analysis

*BF* Breastfeeding, *FF* Formula Feeding Subj. norm = subjective norm ***p < .001, **p < .01, *p < .05

^a^Scotland = reference group

^b^Female = reference group

## Discussion

We found clear differences in infant feeding beliefs and intentions between national groups, with large effect sizes, mirroring differences in breastfeeding rates, showing how young people’s breastfeeding decisions reflect what is culturally normal. The pattern of parenting beliefs, attitudes and intentions was more variable with generally smaller effects. Effect sizes were larger for national differences than for other demographic factors, including age and sex, suggesting breastfeeding beliefs are highly culturally specific. Adolescents’ beliefs in shared parenting of infants reflect cultural changes in men’s and women’s work and family roles [1] and were positive linked with intention to breastfeed. This did not support the hypothesis that young men’s wish to be more involved in feeding predisposes towards use of formula milk. This is potentially important. Two-thirds of adolescents intended to become parents, and shared parenting and gender equality in parenting were endorsed by both sexes and across ages. Linking breastfeeding more clearly with equal parenting by showing how both parents can be involved in parenting whilst the mother is breastfeeding may help to make it more socially normal and desirable for new parents.

Nordic participants were 18 times more likely to intend to breastfeed than those from Scotland and USA. Using comprehensive measures of descriptive and injunctive normative beliefs in a TPB framework highlights the source of these cultural influences. Nordic participants have significantly more exposure to breastfeeding, more positive breastfeeding norms and least exposure to formula feeding. Norms were also highly significant predictors of breastfeeding intention. Social norms from parents and peers are particularly significant influences on infant feeding intention and behaviour in young people [36, 44] and we would expect young people to be more influenced by cultural transmission of norms than adults [45]. Transmission of norms occurs at different levels of reference, including the family, peers community, institutional and policy level. We have no evidence for the relative influence of these, however we would expect experience of infant feeding and parenting practices within their own families to be more salient for young non-parents, since they are unlikely to have been exposed to relevant peer group influences, particularly at younger ages. Our study shows huge cultural differences in the way participants were fed themselves as babies, and more awareness of this (i.e. fewer ‘don’t know) in Nordic participants, indicated more certainty in breastfeeding intentions. Intergenerational discussion of breastfeeding and parenting practices is clearly an important focus for efforts to change social or cultural norms [46]. Nevertheless, the positive relationship we identified between shared parenting and intended breastfeeding is an important potential facilitator of breastfeeding promotion in prospective parents, where an exclusive focus on breastfeeding may seem less relevant (less socially normative), or coercive [47].

Surprisingly there were few overall age and sex differences, and neither were significant predictors of breastfeeding intention. Girls had more positive parenting attitudes. Gender norm scores suggested both parents should be equally involved with caring tasks, although both male and female participants ascribed the main caring role to the mother, and predictably, young men were significantly more likely to think the mother should carry out more tasks, including change diapers, soothe and babysit. Girls also had more exposure to infant feeding generally and more positive breastfeeding norms, as expected.

This study focused only on the decision to initiate breastfeeding. Arguably the role of fathers in supporting breastfeeding may be a more relevant determinant of the *duration* of breastfeeding. More equal and supportive gender roles are linked with feeding longer [28], and the converse it also true [30, 34]. Attitudes may change once a baby is born and the realities of parenthood are more apparent, so it is important to replicate our work with new parents to investigate this relationship further.

### Limitations

There were some methodological issues which may have affected the reliability of results. Using an ‘opt-out’ methodology for recruitment may have been ethically questionable – we could not be certain that parents had received the information, and participants may have felt pressurised to participate in school settings. Nevertheless we would justify our approach here in terms of ensuring adequate sample size and representativeness [48]. The sample sizes and demographic characteristics were unbalanced. The Scottish sample was larger and younger, and there were fewer males in Nordic and USA samples, although we did not find evidence that this biased results. We know that SES is a key variable affecting breastfeeding rates. We measured mother’s education level as a proxy variable for SES, as we anticipated it would be difficult to code cross-national data on occupation reliably. Nevertheless, several participants did not (or could not) provide this information, so missing data was an issue for this variable. Since it was difficult to establish equivalence across samples we did not include this in the final regression model. The omission of ethnicity measurement was less important for our Scottish sample, which has a largely White British composition, than for the other countries. However Texas has larger proportions of Black American and Hispanic groups, and future work should include reliable measurement of ethnicity. We used variable sampling methods, and cannot estimate bias in terms of response rates. Data in schools was collected on a class by class basis, so may have reflected social desirability and teacher’s interest. The social networking approach used in Finland may have incurred socio-economic biases in respondents using particular websites. Since there were several univariate statistical tests carried out with the data, there was also potential for Type 1 errors.

## Conclusions

This was a robust and large-scale cross-cultural survey of attitudes to shared parenting and infant feeding. We found that young peoples’ infant feeding exposure, beliefs and intentions differed according to their country of origin. Young people in Nordic countries were much more likely to intend to breastfeed than those from Scotland and the USA and this was reflected in their beliefs, social norms and confidence. Parenting beliefs were also important predictors of breastfeeding intentions. This suggests practitioners, including health professionals and educators promoting breastfeeding or parenting skills should consider cultural factors when developing interventions. We argue that discussing breastfeeding in the context of parenting choices with young people offers a potentially more helpful focus than simply highlighting health benefits.

## Abbreviations

anova: Analysis of variance
ES: Effect size
OR: Odds ratio
SES: Socio economic status
SN: Subjective norm
TPB: Theory of planned behaviour
UK: United Kingdom
USA: United States of America
